# Torsional and flattening effect on corneal astigmatism after cataract surgery: a retrospective analysis

**DOI:** 10.1186/s12886-017-0399-1

**Published:** 2017-02-08

**Authors:** Yuli Park, Hyun Seung Kim

**Affiliations:** Department of Ophthalmology, Yeouido St. Mary’s Hospital, College of Medicine, The Catholic University of Korea, 62 Yeouido-dong, Yeongdeungpo-gu, Seoul 150-713 Korea

**Keywords:** Steep meridian incision, Cataract surgery, Posterior corneal astigmatism, Shifting of steep axis

## Abstract

**Background:**

To evaluate the torsional and flattening effect of steep meridian incisions and influence of posterior corneal astigmatism (PCA) on total corneal astigmatism (TCA) after cataract surgery.

**Methods:**

One hundred thirty-two eyes underwent cataract surgery with steep meridian 2.2 mm microcoaxial and 2.85 mm conventional clear corneal incisions. Eyes were divided into with-the-rule (WTR) astigmatism and against-the-rule (ATR) astigmatism groups depending on the steeper meridian and measured with autokeratorefractor and Pentacam® before surgery, at 1 day, 1 week, 1 and 2 months postoperatively. Polar vector analysis was used to evaluate torsional effect of steep meridian incisions.

**Results:**

A decrease in astigmatic polar value (AKP) (+0) was observed in both keratometric and total astigmatism (TA) after 1 and 2 months, although the decrease was only statistically significant in TA (*p* < 0.05). The AKP(+45) was more significant in the conventional group than the microcoaxial group at 2 months postoperatively (*p* < 0.05, respectively). There was a significant correlation between corneal thickness of the superior quadrant and PCA in the WTR group (*p* = 0.028). In eyes with anterior corneal astigmatism smaller than 0.55D of WTR astigmatism and PCA greater than 0.35D of WTR astigmatism showed greater shifting of steep axis and also increment of refractive cylinder powers.

**Conclusions:**

In eyes with superior corneal thickness greater than 714.5 μm and PCA greater than 0.35D of WTR astigmatism, steep meridian incision may cause a significant torsional effect and off-steep meridian change, contributing to an increment of postoperative residual manifest astigmatism after cataract surgery.

## Background

Uncorrected astigmatism, even when as low as 1.0 diopter (D), can significantly affect distance and near visual acuity and lead to patient’s dissatisfaction after cataract surgery [1]. The control of corneal astigmatism during cataract surgery has been of increasing importance. More than 30% of patients require astigmatism correction during cataract surgery because of the prevalence of corneal astigmatism of >1.0 D [2]. Therefore, accurate measurement of total corneal astigmatism (TCA) is a critical element in correcting astigmatism during cataract surgery. However, obtaining an exact assessment of corneal astigmatism that would enable accurate surgical planning and perfect correction of astigmatism has been a challenge. Because surgically induced astigmatism (SIA) is a known consequence of creating the incision necessary for cataract surgery [3], the amount of astigmatism to be corrected at the time of surgery must be the vector sum of the preoperative corneal astigmatism and any SIA [3, 4]. Although both the anterior and posterior corneal surfaces contribute to the TCA, TCA was usually derived solely from the keratometer or corneal topography-measured anterior corneal radius. This mathematical shortcut is used because of past difficulties in measuring the posterior corneal surface. The keratometric index was developed so that the omission of the posterior corneal surface measurement could be compensated for by measuring only the anterior corneal surface [5]. However, it has been shown that the traditional keratometric index might lead to a significant error in actual corneal power estimation [6].

Results in several studies indicate that posterior corneal astigmatism (PCA) contributes significantly to the TCA and these studies report posterior astigmatism values of −0.26 to −0.78D [7, 8]. Recent study showed that neglecting the posterior surface measurement may lead to significant deviation in corneal SIA estimation as well as identification of preexisting astigmatism [9, 10]. Study found that for toric intraocular lens (IOL) implantation, ignoring the PCA would result in overcorrection in eyes having with-the-rule (WTR) anterior corneal astigmatism (ACA) and undercorrection in eyes with against-the-rule (ATR) astigmatism [11]. Thus, incorporating PCA into the cataract surgery might improve refractive outcomes. Few studies comprising a large number of patients have been published on astigmatic changes after cataract surgery, though they do not emphasize the posterior component of the corneal astigmatism [8, 9].

Posterior corneal imaging can be carried out with an OrbScan (Bausch and Lomb, Rochester, NY), a Pentacam (Oculus, Inc., Wetzlar, Germany), a Scheimpflug imaging with a Placido disc (Sirius; Costruzione Strumenti Oftalmici, Florence, Italy) or a Placido disc in combination with a dual-channel Scheimpflug camera using ray tracing (Galilei dual Scheimpflug analyzer; Zeimer Group, Port, Switzerland). The Pentacam® is a rotating Scheimpflug camera that images the anterior segment which provides elevation maps of the anterior and posterior corneal surfaces, pachymetry maps and biometric measurements of the anterior segment [12]. It measures 25,000 data-points over the cornea in <2 s and uses a 475-nm monochromatic blue light for imaging, whereas the camera takes images at a resolution of 1392 × 1040 pixels and uses 138,000 true elevation data points over the whole cornea [12].

The purpose of this study was to determine the influence of the preoperative PCA on postoperative TCA using the Pentacam® in patients undergoing phacoemulsification using standard 2.85 and 2.2 mm microcoaxial steep meridian clear corneal incisions. We took posterior cornea surface into consideration and evaluated the effect of steep meridian incisions on TCA in terms of flattening and torsional effect.

## Methods

### Study group

This study was conducted in accordance with the institutional review board regulations (IRB), investigator obligations, and followed the tenets of the Declaration of Helsinki. All patients gave signed informed consent following a discussion of the details of the intervention and the possible risks. This study was reviewed and approved by the Institutional Review Boards (IRBs) of Yeouido St. Mary’s Hospital, College of Medicine, the Catholic University of Korea (IRB#SC13RISI0168).

Retrospectively, this study reviewed consecutive patients who underwent phacoemulsification with IOL implantation and had corneal power measurements with the Pentacam® Scheimpflug imaging system (Oculus, Inc., Wetzlar, Germany) from July 2014 to April 2015. Inclusion criteria were patients with good-quality Scheimpflug analyzer scans, no previous ocular trauma or surgery, no corneal or other ocular diseases, and no contact lens use within 2 weeks of the Pentacam® measurements. Exclusion criteria included patients with irregular astigmatism preoperatively, previous ocular surgery or diseases, diabetic or other systematic disease.

Our study included 132 randomly selected eyes of 82 patients. Eyes were divided into two groups depending on the corneal steep meridian as follows: (1) WTR group with corneal steep meridian at 60 to 120° which included 64 eyes and (2) ATR group with corneal steep meridian at 0 to 30° or 150 to 180° which included 68 eyes. Patients with steep meridians in the superonasal and nasal locations were not included. To establish if any differences detected were present throughout the incision length selected, a comparison of two subgroups of eyes with 2.2 mm sized microcoaxial incisions and 2.85 mm standard incisions were made within the WTR and ATR groups. The keratometric astigmatism was derived from anterior surface measurement using corneal keratometric index (*n* = 1.3375). Cataracts of similar density according to the Lens Opacities Classification System III scale were included.

### Clinical examination

Before cataract surgery, all patients had a comprehensive ophthalmologic examination including manifest refraction and keratometry using an autokeratorefractor (RK-5, Canon, Inc.), the Pentacam® measurements, slit-lamp evaluation, tonometry, and funduscopy through a dilated pupil. In the case of the Pentacam’s ray-tracing technology, corneal power of the anterior surface and posterior surface can be determined at a number of selected distances from either the pupillary center or corneal apex using a ring (perimeter) or zone (the area within a given radius), with the result termed the total corneal refractive power (TCRP). Three millimetre apex/zone data from the Scheimpflug system were used for analysis. Nuclear opalescence was graded using the Lens Opacities Classification System III. Axial length was measured using the optical biometry (IOL Master, Carl Zeiss Meditec AG) or if the optical biometry was unavailable, using the A-scan ultrasonography (Axis-II, Quantel Medical). The axial length of 124 eyes was measured by the optical biometry and that of eight eyes was by the A-scan ultrasonography. Patients’ data extracted from the medical records included demographics, presence of preexisting ocular morbidity, preoperative and postoperative visual acuity, preoperative keratometry, preoperative refraction, indications for phacoemulsification, incision location and size, intraoperative and postoperative complications, diopters of the implanted IOL, postoperative keratometry and refraction. Each patient had clinical examinations at 1 day, 1 week, 1 and 2 months after the cataract surgery.

In this study, we analyzed the data obtained by the Pentacam® before and after cataract surgery. Both anterior and posterior surface measurements were obtained preoperatively, including the degree of flat and steep meridian and radius at these meridians. In the case of image distortion or a lack of any observed data, the image taken was repeated. For the pachymetry, central corneal thickness was recorded, and the peripheral corneal thickness in each quadrant (superior, inferior, nasal, and temporal) was calculated as a mean by the Pentacam®. The measurements were collected between 4 and 7 mm to calculate and average peripheral corneal thickness.

Corneal astigmatism was analyzed using polar value analysis as suggested by Naeser [13]. Because refractive data in the form of sphere, cylinder and axis are unsuitable for mathematical analysis, the polar value system was utilized for all calculations [13]. Preoperative and postoperative astigmatism were transformed to orthonormal astigmatism polar values [AKP(0), AKP(+45)]. SIA with preoperative astigmatism M1@α and postoperative astigmatism M2@β was calculated as follows:

$$ \mathrm{A}\mathrm{K}\mathrm{P}{\left(+0\right)}_{\mathrm{pre}} = \mathrm{M}1,\ \mathrm{A}\mathrm{K}\mathrm{P}{\left(+45\right)}_{\mathrm{pre}} = 0; $$

$$ \mathrm{A}\mathrm{K}\mathrm{P}{\left(+0\right)}_{\mathrm{post}} = \mathrm{M}2*\left[ \sin 2\left(\upbeta +90-\upalpha \right) - \cos 2\left(\upbeta +90-\upalpha \right)\right], $$

$$ \mathrm{A}\mathrm{K}\mathrm{P}{\left(+45\right)}_{\mathrm{post}} = \mathrm{M}2*\left[ \sin 2\left(\upbeta +45-\upalpha \right) - \cos 2\left(\upbeta +45-\upalpha \right)\right]; $$

$$ \varDelta \mathrm{A}\mathrm{K}\mathrm{P}\left(+0\right) = \mathrm{A}\mathrm{K}\mathrm{P}{\left(+0\right)}_{\mathrm{post}} - \mathrm{A}\mathrm{K}\mathrm{P}{\left(+0\right)}_{\mathrm{pre}},\ \varDelta \mathrm{A}\mathrm{K}\mathrm{P}\left(+45\right) = \mathrm{A}\mathrm{K}\mathrm{P}{\left(+45\right)}_{\mathrm{post}} - \mathrm{A}\mathrm{K}\mathrm{P}{\left(+45\right)}_{\mathrm{pre}} $$

In this transformation, the steeper preoperative meridian was consistently used as a reference, in order to analyze corneal flattening effect as well as torsional effect induced by surgery. The surgically induced polar value in the meridian C0[∆AKP(C0)] was the meridional power causing a decrease or increase in power along that plane. The surgically induced polar value in the meridian (C45)[∆AKP(C45)] was the torsional force twisting the astigmatism in a counterclockwise or clockwise direction.

### Surgical technique

One surgeon performed all cataract surgeries under topical anesthesia which was induced with 0.5% paracaine hydrochloride (Alcaine®, Alcon, Puurs, Belgium). Before surgery, the eye was prepared and draped using sterile techniques. The same tip angulation was used for all patients. The meridian of corneal incision was chosen by rounding the steep corneal meridian to the nearest 10°. The 2.2 mm or 2.85 mm sized metal keratome was used for clear corneal incisions on the steeper corneal meridian, which was predetermined by the Pentacam®. Phacoemulsification was performed with a side port of a 1 mm 70-degree incision away from the main incision. Cataracts were removed using 0.9 mm 30° bevel Kelman tips and the phacoemulsification settings for both groups were 250 mmHg vacuum, and an aspiration rate of 30 cm^3^/min. The range of phacoemulsification time was 0.5 to 1 min and torsional mode (OZil mode, Alcon, Fort Worth, TX, USA) was selected. A foldable IOL was inserted in the posterior chamber thereafter. At the end of surgery, the corneal wound was hydrated with balanced salt solution, and no suture was applied. There was no intraoperative complication in all cases. After surgery, all patients instilled 0.5% moxifloxacin (Vigamox^Ⓡ^, Alcon, Fort Worth, TX, USA) and 1% prednisolone acetate (Pred Forte®, Allergen, Irvine, CA, USA) eye drops four times daily for 1 week then, slowly tapered.

### Statistical analysis

Statistical analysis was performed with SPSS version 13.0 (SPSS, Chicago, Illinois, USA). Shapiro-Wilk test was performed to test the normality distribution of all data. Because the refractive components are correlated, multivariate statistical analysis using the Hotelling trace test was used for comparison of intraindividual changes. To establish if the magnitude of astigmatic error for with-the-rule and against-the-rule eyes represented real cylindrical power error, one-sample *t* tests were used. Univariate analysis was used to examine changes in respective polar values [ΔAKP(0), ΔAKP(+45)]. All postoperative parameters were compared to preoperative ones. The Spearman rank test was applied to explore the correlations. *P* values 0.05 or less were considered to be statistically significant.

## Results

Our study included 132 randomly selected eyes of 82 patients. The median age of our patients was 65.19 ± 10.84 years and 45 of the patients (54.88%) were woman. The mean nuclear opalescence scores were 3.83 ± 1.3 in the microcoaxial cataract surgery (MCCS) group and 3.79 ± 1.5 in the conventional cataract surgery (CCS) group (*p* = 0.382). The mean nuclear color scores were 3.57 ± 1.29 in the MCCS group and 3.61 ± 1.08 in the CCS group (*p* = 0.403). The mean preoperative and postoperative spherical equivalents were −0.51 ± 3.74D and −0.05 ± 0.85D, respectively. The mean preoperative PCA was a median of 0.29 D and exceeded 0.5 D in 10.71%, 0.75 D in 2.14%, and 1.0 D in 0.06%. The preoperative keratometry and other data are presented in detail in Table 1.

**Table 1:** Patient demographics

Parameter	WTR group	ATR group	*p* value
Eyes (n)	64	68	.
MCCS: CCS (n)	33:31	35:33	.
Sex (M:F) (n)	18/23	19/22	0.082
Mean age (years)	60.94 ± 9.42	69.58 ± 10.31	0.305
Laterality (OD:OS) (n)	34/30	33/35	0.594
Preoperative mean K (D)	44.3 ± 1.2	43.8 ± 1.3	0.296
Mean IOL power (D)	19.4 ± 3.9	20.2 ± 3.5	0.658
CCT (μm)	541.8 ± 30.9	542.3 ± 32.6	0.874
ACD (mm)	3.51 ± 0.08	3.09 ± 0.12	0.182
Axial length (mm)	23.31 ± 2.07	22.29 ± 1.98	0.539
Endothelial cell count	2832.51 ± 315.64	2791.29 ± 332.91	0.377
SE (D)	−1.51 ± 0.35	−1.62 ± 0.51	0.571
UCVA (log MAR)	0.62 ± 0.31	0.55 ± 0.29	0.365
BCVA (log MAR)	0.29 ± 0.11	0.27 ± 0.12	0.834
IOP (mmHg)	14.3 ± 2.51	14.6 ± 2.80	0.341

Values are presented as mean ± SD

*ACD* anterior chamber depth, *ATR* against-the-rule, *BCVA* best corrected visual acuity, *CCS* conventional cataract surgery, *CCT* central corneal thickness, *IOL* intraocular lens, *IOP* intraocular pressure, *K* keratometry, *KA* keratometric astigmatism, *MCCS* microcoaxial cataract surgery, *SE* spherical equivalent, *TA* total astigmatism, *UCVA* uncorrected visual acuity, *WTR* with-the-rule

### Corneal astigmatism and thickness

The orientation of the ACA was WTR in 48.48% (*n* = 64), ATR in 51.52% (*n* = 68). Regarding the posterior astigmatism, it was WTR in 94.49% (*n* = 125), ATR in 5.51% (*n* = 7).

Table 2 shows preoperative pachymetry data and their correlation with PCA. The preoperative peripheral corneal thickness was significantly greater in the superior quadrant than in the nasal, inferior and temporal quadrants in WTR group. There was a significant correlation between corneal thickness of the superior quadrant and PCA in the WTR group (*r* = 0.516, *p* = 0.028), but not in the ATR group. There was no significant difference in peripheral corneal thickness of the nasal, temporal and inferior quadrants in both WTR and ATR group.

**Table 2:** Correlations between corneal thickness and PCA

Group	Parameters	Mean ± SD	*p v*alue
WTR group	CCT	541.8 ± 30.9	0.143
	PCT (Superior)	631.2 ± 34.3	0.028 (*r* = 0.516)^a^
	PCT (Inferior)	619.6 ± 31.2	0.529
	PCT (Nasal)	608.3 ± 32.4	0.420
	PCT (Temporal)	609.9 ± 30.5	0.396
ATR group	CCT	542.3 ± 32.6	0.164
	PCT (Superior)	615.2 ± 33.2	0.541
	PCT (Inferior)	608.4 ± 32.1	0.368
	PCT (Nasal)	599.8 ± 32.4	0.649
	PCT (Temporal)	613.4 ± 30.5	0.113

Values are presented as mean ± SD

*ATR* against-the-rule, *CCT* central corneal thickness, *PCA* posterior corneal astigmatism, *PCT* peripheral corneal thickness, *WTR* with-the-rule

^a^ Correlation between corneal thickness and posterior corneal astigmatism using the Pearson correlation coefficient

### Surgically induced astigmatism

**Fig. 1:**
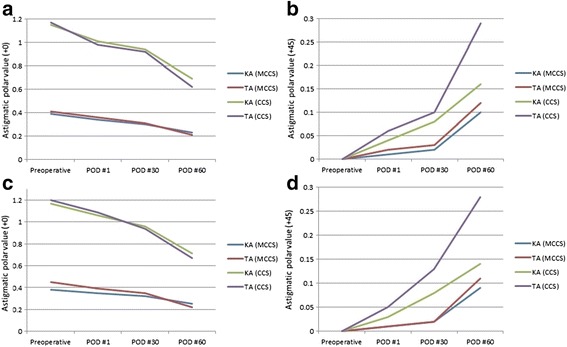
Polar value analysis of keratometric and total astigmatism in WTR (**a**, **b**) and ATR group (**c**, **d**). (ATR = Against-the rule; CCS = Conventional cataract surgery; KA = keratometric astigmatism; MCCS = Microcoaxial cataract surgery; TA = Total astigmatism; WTR = With-the-rule)

Preoperative keratometric astigmatism (KA) magnitude ranged from 0.25 D to 2.21 D. The preoperative posterior surface astigmatism had an arithmetic mean of 0.29 D in our study. Figure 1 represents the polar value analysis of keratometric and total astigmatism in the WTR and ATR group at 1 day, 1 week, 1 and 2 months postoperatively. In the WTR group, the ∆AKP(+0) of total astigmatism (TA) changed significantly after 1 and 2 months, while no significant changes were observed in KA and TA after 1 day postoperatively as well as in KA after 1 and 2 months (Table 3). In the ATR group, the ∆AKP(+0) of TA changed significantly at 1 and 2 months postoperatively, while no significant changes were revealed in KA and TA at 1 day postoperatively as well as in KA after 1 and 2 months. Decrease in AKP(+0) was observed in both keratometric and total astigmatism at 1 and 2 months after cataract surgery, although univariate analysis showed that the decrease was not statistically significant in keratometric astigmatism in both WTR and ATR group (Table 3).

**Table 3:** Polar value analysis of astigmatism in WTR and ATR group after cataract surgery

			1 month postoperatively				2 months postoperatively			
			∆AKP(+0)	*p* value	∆AKP(+45)	*p* value	∆AKP(+0)	*p* value	∆AKP(+45)	*p* value
WTR	MCCS	KA	−0.09 ± 0.25	0.514	0.02 ± 0.01	0.769	−0.15 ± 0.21	0.312	0.10 ± 0.07	0.658
		TA	−0.12 ± 0.26	0.026	0.03 ± 0.01	0.115	−0.22 ± 0.20	0.016	0.12 ± 0.10	0.012
	CCS	KA	−0.23 ± 0.81	0.122	0.08 ± 0.04	0.281	−0.47 ± 0.61	0.310	0.16 ± 0.13	0.184
		TA	−0.27 ± 0.80	0.017	0.1 ± 0.05	0.046	−0.55 ± 0.63	0.008	0.29 ± 0.21	0.009
ATR	MCCS	KA	−0.07 ± 0.23	0.438	0.02 ± 0.01	0.637	−0.13 ± 0.19	0.306	0.09 ± 0.08	0.715
		TA	−0.11 ± 0.29	0.041	0.02 ± 0.01	0.790	−0.20 ± 0.21	0.026	0.11 ± 0.10	0.038
	CCS	KA	−0.21 ± 0.85	0.118	0.08 ± 0.04	0.101	−0.44 ± 0.86	0.107	0.14 ± 0.11	0.091
		TA	−0.25 ± 0.92	0.011	0.13 ± 0.04	0.024	−0.53 ± 0.89	0.005	0.28 ± 0.19	0.013

Values are presented as mean ± SD

*∆* change, *AKP* astigmatic polar value, *ATR* against-the-rule, *CCS* conventional cataract surgery, *KA* keratometric astigmatism, *MCCS* microcoaxial cataract surgery, *TA* total astigmatism, *WTR* with-the-rule

In the WTR group, univariate analysis revealed a significant change of 0.12 ± 0.10D in ∆AKP(+45) of TA in the MCCS group, 0.29 ± 0.21D in the CCS group at 2 months postoperatively (*p* = 0.012, *p* = 0.009, respectively). In the ATR group, univariate analysis also revealed a significant change of 0.11 ± 0.10D in ∆AKP(+45) of TA in the MCCS group, 0.28 ± 0.19D in the CCS group at 2 months postoperatively (*p* = 0.038, *p* = 0.013, respectively) (Table 3). The univariate analysis showed that the AKP(+0) in each incision group decreased significantly and the AKP(+45) of TA, which is a torsional force twisting in the astigmatic direction, was also significant at 2 months postoperatively. The magnitude of AKP(+45) was significantly lower in the MCCS group than in the CCS group, indicating less surgical induced astigmatism in the MCCS group.

**Fig. 2:**
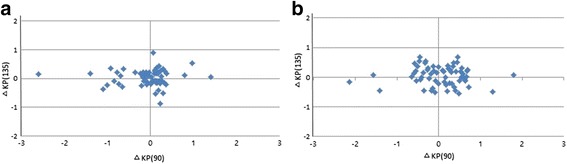
Difference of surgically induced astigmatism between instruments expressed as pairs of polar values. Autokeratometry versus Scheimpflug imaging in MCCS (**a**) and CCS group (**b**) at postoperative 2 month period

The net astigmatism obtained from each of the two instruments was converted to polar values in the form of KP(90) and KP(135). The differences in KP(90) and KP(135) between the incision length selected were calculated and plotted (Fig. 2).

### Shifting of axis

The shifting of steep axis was confined to those eyes having small ACA with relatively large PCA in the WTR group which showed the against-the-rule shift of varying degree at 2 months postoperatively. In eyes with ACA smaller than 0.55D of WTR astigmatism and PCA greater than 0.35D of WTR astigmatism showed greater shifting of steep axis and also increment of refractive cylinder powers in the WTR group (Table 4). The peripheral corneal thickness was significantly greater in the superior quadrant than in the temporal quadrant in the eyes with greater PCA of WTR astigmatism in the WTR group. Axis deviation was calculated as the difference between postoperative and preoperative values. Stabilization of axis deviation which was shifted at 1 day postoperatively, occurred after 1 week and showed similar results at 1 and 2 months postoperatively. The amount of shifted axis and the increment of the refractive cylinder power were significantly greater in the CCS group than the MCCS group at 2 months postoperatively.

**Table 4:** Comparision of factors in patients who showed shifting of steeper axis postoperatively in WTR group

		Unshifted group	Shifted group	*p v*alue
Eyes (n)		48	16	.
Age (years)		64.8 ± 10.06	55.4 ± 9.18	0.125
Postoperative steeper axis		97.3 ± 10.1	5.4 ± 4.2	0.003
MCCS (n)		31	5	0.019
CCS (n)		17	11	0.346
Postoperative change of refractive cylinder (D)		- 0.54 ± 0.27	0.43 ± 0.26	0.017
Preoperative corneal thickness (μm)	Superior	622.4 ± 31.5	714.5 ± 35.2	0.008
	Temporal	620.8 ± 32.9	617.5 ± 31.6	0.692
Preoperative corneal astigmatism (D)	Anterior	1.16 ± 0.38 @ 91.73	0.55 ± 0.13 @ 93.84	0.007
	Posterior	0.19 ± 0.08 @ 92.56	0.35 ± 0.09 @ 91.51	0.005
	Total	1.04 ± 0.26 @ 91.92	0.29 ± 0.08 @ 93.18	0.010

Values are presented as mean ± SD

*CCS* conventional cataract surgery, *MCCS* microcoaxial cataract surgery, *WTR* with-the-rule

## Discussion

Corneal astigmatism after phacoemulsification surgery depends on the location, configuration and size of cataract incision, presence or absence of wound suture, optical center of the cornea, and surgical approach [14]. Superior, superotemporal, temporal and steep axis location incisions are commonly used by several surgeons [15]. Due to the population tendency for the steep meridian of the posterior cornea to be aligned vertically, a negative power effect on corneal power at or near the vertical meridian exists. In eyes with with-the-rule corneal astigmatism, an overestimate of corneal astigmatism will result from ignoring this effect. Conversely, corneal astigmatism in against-the-rule corneal curvature will be underestimated.

In modern cataract surgery, the trend is to minimize surgical trauma, which means efforts are made to reduce incision size, reduce ultrasound power and improve efficiency in order to reduce SIA and endothelial cell injury. Smaller incisions result in less invasive and safer surgery, better intraoperative stability of the anterior chamber, less postoperative intraocular inflammation, fewer incision-related complications, reduced SIA, faster and better postoperative visual recovery, and good postoperative refractive outcomes [15]. It was generally accepted that smaller incision size is one of the determining factors for less SIA [16]. Luo et al. compared temporal location at 1.8, 2.2, 3.0 mm clear corneal incision sizes [17]. In their study, the mean SIA in the 1.8 and 2.2 mm groups was significantly less than that in 3.0 mm group after 1 month, without a significant difference between the 1.8 and 2.2 mm groups. Hayashi et al. showed that SIA at 2 months postoperatively was 0.74D after coaxial 2.65 mm small-incision cataract surgery and 0.56 D after 2.0 mm microincision cataract surgery [18]. Wilczynski et al. compared SIA at 1 month after coaxial phacoemulsification through a 1.8 mm microincision with that for bimanual phacoemulsification through a 1.7 mm microincision [19]. The results showed mean SIA of 0.42 ± 0.29D for the coaxial MICS group and 0.50 ± 0.24 D for the bimanual group and the difference was not statistically significant. Our study demonstrated that torsional effect was significantly lower in the 2.2 mm MCCS group than in the 2.85 mm CCS group which showed similar results as previous studies above.

A significant decrease in AKP(0) was observed only in TCA of the WTR and ATR group at 1 and 2 months postoperatively. This indicated the flattening effect of steep meridian incision, which reduced the preexisting astigmatism along the meridian of incision. In the study by Borasio et al., steep meridian incision induced about 0.63 D flattening effect along the meridian [20]. In our study, the mean flattening effect was from 0.13 D to 0.22 D in MCCS group and from 0.44 D to 0.55 D in CCS group at different follow-up time. However, this flattening magnitude was lower in the MCCS group than the results reported by Borasio et al., but similar in the CCS group [20].

Several studies have evaluated pachymetry mapping and demonstrated that the corneal thickness profile is not uniform depending on the radial direction [21]. Such nonuniformity of corneal thickness distribution influences the anterior-to-posterior ratio of corneal curvature and jeopardizes the validity to calculate corneal refraction using the keratometric index. Especially if the anterior and posterior corneal curvatures lack mutual linearity, estimation of PCA and KA based on the measurements of anterior corneal curvature alone can be misleading. Although previous studies have investigated central corneal thickness, there has been a paucity of information on peripheral corneal thickness [22]. Recently, development of the Scheimpflug camera has led to more precise and easier measurements of peripheral corneal thickness, which allow detailed assessments [23–26]. In this study, we compared peripheral corneal thickness in the four quadrants and discussed the difference in the WTR and ATR group undergoing cataract surgery. We found that in eyes with greater PCA, the cornea was thicker in the superior direction than in the temporal and nasal direction in the WTR group, which can explain why the posterior cornea surface became more ATR astigmatic. In these eyes, the clear corneal incision made on the steeper corneal meridian resulted in flattening effect of steeper meridian, but also torsional effect on the other meridian that showed unwanted postoperative astigmatism.

Keratometric power is calculated using the keratometric index and anterior corneal curvature on the basis of the premise that the corneal thickness profile is uniform. The posterior corneal curvature is thus presumed to have a constant and linear relationship with the anterior corneal curvature. However, we found that the magnitude of vector difference between the KA and TA was significantly correlated with the difference in the PCA especially in the WTR group who had greater peripheral corneal thickness in the superior quadrant. This may explain why the keratometric astigmatism misinterpreted TCA in our study.

Our study found a significant change between preoperative and postoperative TCA at 1 and 2 months postoperatively (*p* < 0.05). One possible source for this change may be a significant change in AKP(+45) of TCA after 1 and 2 months. A change in AKP(+45) indicated a clockwise or counterclockwise torsion in the direction of the steep meridian of astigmatism. In our study, a mean change of about 0.29 ± 0.21D in the CCS group of the WTR group indicated a torsion that may be not only statistically significant but also clinically significant. The shifting of steep axis was confined to those eyes having small ACA with relatively greater PCA in the WTR group.

It is well known that the horizontal meridian is approximately 1 mm wider than vertical length. A superior incision is closer to the corneal apex than a temporal incision could explain the greater effect of incision location on the central corneal curvature making greater torsional effect and steeper axis shifting which is revealed in our study. In addition, in superior incision, the presence of the upper eyelid may play a role by blinking, resulting in wound distortion and stretch of the cornea during wound healing after cataract surgery which could affect the total corneal astigmatism than the temporal incision.

Several reasons may account for unintended torsion after cataract surgery. First, an incision not actually placed on the steeper meridian, may have occurred due to difficulty in performing it in deep-set eyes. An off- steep meridian incision may occur due to inaccurate mark before surgery, or difficulty in performing steep meridian incision in eyes with oblique astigmatism. In our study, we marked the cornea at the slit-lamp before anesthesia to avoid the cyclotorsion from seated to supine position. During the surgery, a circular axis marker was used to locate the steeper meridian. This increased the accuracy in our performing the incision on steep meridian. Second, an error in determining steeper meridian of preoperative astigmatism may have contributed to the torsional effect. However, previous study revealed satisfactory accuracy of the Pentacam® in measuring preoperative astigmatism, and we used it, which enabled us to have accurate identification of the steeper meridian [27]. Finally, the PCA may contribute to the off-steep-meridian change induced by the steep meridian incision as no statistically significant off-steep-meridian change was observed in terms of KA, which was calculated by neglecting posterior corneal surface measurements. The steep meridian incision determined by steep meridian of anterior surface may induce a significant off-steep meridian change in posterior cornea surface, thus contributing to a torsional effect in TCA.

The vector mean of the preoperative PCA was similar to the vector mean of the postoperative residual manifest astigmatism in our study. This suggests that the occult PCA remains untreated when the automated keratometer is used to measure preoperative TCA. In previous study, using devices that calculate TCA based on anterior corneal surface only, WTR astigmatism was overestimated by 0.5 to 0.6 D and ATR astigmatism was underestimated by 0.2 to 0.3 D [3]. This shows that the TCA prediction errors from these devices were primarily caused by the PCA as in our study. Clinically, this implies that the cylinder power of toric IOLs has a trend to be overestimated in WTR eyes and underestimated in ATR eyes when the calculation is only based on measurements of the anterior cornea. Therefore, toric IOL nomogram accounting for PCA should be used.

The surgeon should consider selecting the location and shape of the incision for preoperative corneal shapes in which the back-surface astigmatic shape matches that of the front surface and is of significant magnitude. Conversely, the surgeon should also consider selecting the location and shape of the incision for preoperative corneal shapes in which the back surface astigmatic shape is the opposite of the front surface and is of significant magnitude. In our study, taking into account the mean values of PCA, we concluded that it might be important to leave eyes with small amounts of with-the-rule refractive astigmatism in young patients. Because this may allow for the ongoing against-the-rule shift that occurs in most eyes as patients get older.

Limitations of this study include the retrospective design and a relatively small sample. But, preoperative KA and posterior surface astigmatism distribution did not differ much from that of cataract candidate distribution. Further evaluation on a greater number of patients is advisable and further study would benefit from the allowance for the against-the-rule shift on the principle that might be universal and of varying degree, patient to patient. In a study of the time required for changes in induced astigmatism to stabilize, Masket et al. found that the cornea stabilized within 2 weeks after surgery when incisions smaller than 3.0 mm were used [28]. Further longer-term studies of the astigmatism might prove helpful in predicting post-operative astigmatism after phacoemulsification.

To our knowledge, no study has evaluated the effect of steep meridian clear corneal incision on TCA when taking posterior surface measurement into account in microcoaxial and conventional cataract surgery. Our study evaluated patients who underwent steep meridian clear corneal incisions during phacoemulsification according to the incision size. When posterior surface astigmatism was considered, steep meridian incisions reduced preoperative astigmatism along the steep meridian but, steep meridian incision may also lead to a significant torsional effect on corneal astigmatism, which could be neglected from routine keratometric astigmatism measurements resulting in shifting of steep axis and also increment of refractive cylinder powers.

## Conclusions

We found that patients with the greater posterior corneal with-the-rule astigmatism had greater corneal thickness in the superior quadrant, making the posterior cornea more ATR astigmatic. Therefore, in eyes with superior corneal thickness greater than 714.5 μm and PCA greater than 0.35D of WTR astigmatism, steep meridian incision may cause a significant torsional effect and shifting of the steeper axis, contributing to an increment of refractive cylinder powers after cataract surgery. The magnitude of torsional effect was lower in the MCCS than in the CCS, indicating less SIA in the MCCS. When the patient shows small TCA on the preoperative examinations, MCCS should be firstly considered rather than CCS to leave less postoperative astigmatism. We hope these findings will add some helpful information to clinical practice in cataract surgery.

## Abbreviations

ACD: Anterior chamber depth
AKP: Astigmatic polar value
ATR: Against-the-rule
BCVA: Best corrected visual acuity
CCS: Conventional cataract surgery
CCT: Central corneal thickness
IOL: Intraocular lens
IOP: Intraocular pressure
K: Keratometry
KA: Keratometric astigmatism
MCCS: Microcoaxial cataract surgery
PCA: Posterior corneal astigmatism
PCT: peripheral corneal thickness
SE: Spherical equivalent
TA: Total astigmatism
UCVA: Uncorrected visual acuity
WTR: With-the-rule
